# Childhood maltreatment is associated with cortical thinning in people with eating disorders

**DOI:** 10.1192/j.eurpsy.2023.914

**Published:** 2023-07-19

**Authors:** M. Battipaglia, N. Attianese, S. Donato, R. Ceres, G. Cascino

**Affiliations:** 1 Department of Psychiatry, University of Campania “Luigi Vanvitelli”, Naples; 2 Department of Medicine, Surgery and Dentistry “Scuola Medica Salernitana”, Section of Neurosciences, University of Salerno, Salerno, Italy

## Abstract

**Introduction:**

Childhood maltreatment (CM) is recognized as non-specific risk factor for the onset of various psychiatric disorders and is associated with a greater severity in their clinical presentation and poorer treatment outcome. These data suggest that maltreated people with eating disorders (ED) may be biologically other than clinically different from non-maltreated people.

**Objectives:**

Aim of the present study was to investigate cortical thickness (CT), a possible biomarker of neurodevelopment, in people with ED with or without history of CM and in healthy women.

**Methods:**

Study participation was proposed to patients consecutively admitted to the adult ED outpatient centre of the University of Salerno. Twenty-four healthy women, 26 with anorexia nervosa (AN) and 24 with bulimia nervosa (BN) underwent a 3T MRI scan. All the participants completed the short form of the Childhood Trauma Questionnaire (CTQ). All neuroimaging data were processed by FreeSurfer. Maps of CT were computed in order to perform a vertex-by-vertex analysis. CT maps underwent a general linear model analysis to evaluate differences among groups. Age and body mass index (BMI) were included as nuisance covariates.

**Results:**

Based on CTQ cut-off scores, 12 participants with AN and 12 with BN were identified as maltreated and 14 participants with AN and 12 with BN as non-maltreatment. All healthy women were “non-maltreated”. Therefore, participants were split in 3 groups: 26 maltreated participants with ED, 24 non-maltreatment participants with ED and healthy control (HC). Compared to HC, maltreated people with ED showed lower CT values in the left rostral anterior cingulate gyrus, while compared to non-maltreatment people with ED showed lower CT values in the left superior frontal, in right caudal middle frontal and in right superior parietal gyri. No significant differences emerged in CT measures between HC and non-maltreatment people with ED.

**Conclusions:**

Present findings show for the first time that in adult people with ED childhood maltreatment is associated with cortical thinning in areas implicated in the modulation of brain processes that are acknowledged to play a role in the psychopathology of ED.

**Disclosure of Interest:**

None Declared

